# Resolving the Phylogeny of the Olive Family (Oleaceae): Confronting Information from Organellar and Nuclear Genomes

**DOI:** 10.3390/genes11121508

**Published:** 2020-12-16

**Authors:** Julia Dupin, Pauline Raimondeau, Cynthia Hong-Wa, Sophie Manzi, Myriam Gaudeul, Guillaume Besnard

**Affiliations:** 1 Laboratoire Evolution & Diversité Biologique (EDB, UMR 5174), CNRS/IRD/Université Toulouse III, 118 Route de Narbonne, 31062 Toulouse, France; julia.guedes-rocha-dupin@univ-tlse3.fr (J.D.); pauline.raimondeau@univ-tlse3.fr (P.R.); sophie.manzi@univ-tlse3.fr (S.M.); 2 Claude E. Phillips Herbarium, Delaware State University, 1200 N. Dupont Hwy, Dover, DE 19901-2277, USA; chwa@desu.edu; 3 Institut de Systématique Evolution Biodiversité (ISYEB), Muséum National d’Histoire Naturelle, CNRS, Sorbonne Université, EPHE, Université des Antilles, 57 rue Cuvier, CP39, 75005 Paris, France; myriam.gaudeul@mnhn.fr

**Keywords:** herbarium, museum collection, mitochondrial DNA, plastome, nuclear ribosomal DNA, phytochromes, low-copy genes, taxonomy, polyploidy

## Abstract

The olive family, Oleaceae, is a group of woody plants comprising 28 genera and ca. 700 species, distributed on all continents (except Antarctica) in both temperate and tropical environments. It includes several genera of major economic and ecological importance such as olives, ash trees, jasmines, forsythias, osmanthuses, privets and lilacs. The natural history of the group is not completely understood yet, but its diversification seems to be associated with polyploidisation events and the evolution of various reproductive and dispersal strategies. In addition, some taxonomical issues still need to be resolved, particularly in the paleopolyploid tribe Oleeae. Reconstructing a robust phylogenetic hypothesis is thus an important step toward a better comprehension of Oleaceae’s diversity. Here, we reconstructed phylogenies of the olive family using 80 plastid coding sequences, 37 mitochondrial genes, the complete nuclear ribosomal cluster and a small multigene family encoding phytochromes (*phyB* and *phyE*) of 61 representative species. Tribes and subtribes were strongly supported by all phylogenetic reconstructions, while a few Oleeae genera are still polyphyletic (*Chionanthus, Olea, Osmanthus, Nestegis*) or paraphyletic (*Schrebera*, *Syringa*). Some phylogenetic relationships among tribes remain poorly resolved with conflicts between topologies reconstructed from different genomic regions. The use of nuclear data remains an important challenge especially in a group with ploidy changes (both paleo- and neo-polyploids). This work provides new genomic datasets that will assist the study of the biogeography and taxonomy of the whole Oleaceae.

## 1. Introduction

The olive family (Oleaceae) is a medium-sized group of woody plants comprising 28 genera and ca. 700 species, distributed on all continents (except Antarctica) in both temperate and tropical environments [1]. Most species are trees, but there are also one herbaceous plant (*Dimetra craibiana*), small shrubs (e.g., *Menodora* spp.) and a few lianas (e.g., *Jasminum* spp., *Chionanthus macrobotrys*). Many Oleaceae species are of economic importance, for the production of oil and fruits (olive), timber (e.g., ash trees), as well as ornaments and fragrances (e.g., jasmines, osmanthuses, lilacs, etc). Moreover, Oleaceae are important components of temperate and tropical ecosystems, with, for example, several species producing drupes and palatable leaves as a common food source to wild animals. Individual species can also support a large number of other organisms, for example, nearly 1000 species (e.g., fungi, insects, birds) are known to be associated with *Fraxinus excelsior* [2].

Oleaceae is currently divided into five tribes, Myxopyreae, Jasmineae, Forsythieae, Fontanesieae and Oleeae, the latter being subdivided into four subtribes (Oleinae, Fraxininae, Ligustrinae, and Schreberinae) [3]. The natural history of the group is not completely understood yet, but its diversification seems to be associated with a few events of polyploidisation (in particular a major event of whole genome duplication in the ancestor of Oleeae) [4,5,6,7,8,9] and the evolution of various reproductive and dispersal strategies [3]. This family thus presents a substantial diversity of flowers (e.g., [1,10,11]) and fruits (e.g., capsules, samaras, drupes) [3,12,13,14]; associated with different vectors of pollination and seed dispersal. Variable breeding systems were also described, from hermaphroditism to dioecy, with several stages often considered as intermediate such as polygamy and androdioecy (e.g., [10,15,16,17,18]). In addition, a di-allelic self-incompatibility system, associated with distyly in some groups, has been reported for a number of species belonging to different tribes (i.e., Myxopyreae [15], Jasmineae [19,20], Forsythieae [21], and Oleeae [1,22,23]).

To better understand trait evolution and patterns of diversification in this group, or to resolve any lingering taxonomical issues, as in the case of the paleopolyploid tribe Oleeae [3,24,25,26], a robust phylogenetic hypothesis is then required. Oleaceae’s systematics has evolved from being based on morphological, cytological, and biochemical traits (e.g., [4,14,27]), to the use of molecular phylogenies during the last two decades. Such advances, though, have mainly consisted in studies that focused on specific groups or partially resolved phylogenetic trees [11,26,28,29,30,31,32,33,34,35,36], and not on the whole family (but see [3,37]). This has been mainly due to difficulties to sample all main Oleaceae lineages and to take into account variable ploidy levels [31]. Recent developments in genomics and museomics present new opportunities to tackle such obstacles, though. Several Oleaceae nuclear and cytoplasmic genomes, as well as transcriptomes have been released [31,38,39,40,41,42,43]. Also herbarium samples, previously deemed unusable, are now accessible allowing for a more comprehensive sampling, and the inclusion of rare, or recently extinct species [31,44,45,46].

Another product of the recent advances in genomics is the possibility to use various, independent genomic regions to reconstruct phylogenies of plant groups. The genome skimming approach, for instance, has allowed for cost-effective sequencing of high-copy fractions of total genomic DNA, such as organellar genomes, and nuclear ribosomal DNA, but it can also generate data sets for low-copy nuclear genes [47,48]. With such a diversity of genomic datasets, one can compare the phylogenetic hypothesis estimated using plastid, mitochondrial and nuclear data and increment the estimation of species trees from gene trees. Recent studies that did such comparisons include determining the origin of wild octoploid species [49], the placement of the Celastrales-Oxalidales-Malpighiales (COM) clade within Rosidae [50], and the origin and evolution of species in *Ludwigia* sect. *Macrocarpon* of Onagraceae [51].

Here, we reconstructed phylogenies of the olive family using protein-coding sequences for 80 plastid coding sequences, 37 mitochondrial genes, the nuclear ribosomal cluster and one small multigene family encoding phytochromes (*phyB* and *phyE*) of 61 representative species to document any patterns of incongruence between datasets, and discuss these in the context of the evolution of Oleaceae. The use of nuclear data remains, however, an important challenge especially in a group with frequent ploidy changes (both paleo- and neo-polyploids). Due to paleo-events of polyploidisation, the basic chromosome number varies among tribes (i.e., *x* = 23 in Oleeae, 14 in Forsythieae, 13 in Fontanesieae, 11 to 13 in Jasmineae, and 11 in Myxopyreae [3,4,6]. As the consequence of whole genome duplication(s), some nuclear genes could be duplicated in polyploid lineages (e.g., Oleeae), and their orthology has to be verified before using them for inferring species phylogenies. In addition, gene duplicates as well as their pseudogenes may inform us on the polyploids ancestors. Reconstructing the phylogeny of multigene families is the first step to identify gene orthologs that could be used for species phylogenetic reconstruction. Here, we chose the closely related phytochrome genes *phyB* and *phyE*, because these low-copy genes have been frequently used for inferring phylogenetic relationships in several plant families (e.g., [52,53]). All these new datasets will not only assist on the study of Oleaceae’s taxonomy, but also its biogeography.

## 2. Materials and Methods

### 2.1. Taxon Sampling and Sequencing

In this study, we sampled a total of 65 species: 61 belonging to the ingroup (Table S1) and four representing outgroups (Table S2). The ingroup included species representing all currently recognized tribes, subtribes and genera in Oleaceae. For such list, we followed the current checklist of accepted taxa in Oleaceae that has been reviewed by the staff at Royal Botanic Gardens (Kew), as part of the project “World Checklist of Selected Plant Families” [54], and the most recent literature (e.g., [32,55]). The outgroup comprised two species also in the Lamiales order, *Avicennia marina* (Acanthaceae) and *Sesamum indicum* (Pedaliaceae), and two species in the Solanales order, *Capsicum annuum* and *Solanum lycopersicum* (both in Solanaceae).

Whole genome sequences (’genome skims’) were obtained for the 61 Oleaceae species. Twenty-two samples were removed from herbarium collections specimens (Table S1). Forty-one accessions were already characterized from previous works [31,42], and we newly analyzed 20 species belonging to Jasmineae (six species, three genera), Myxopyreae (four species, three genera), Forsythieae (*Abeliophyllum*), and Oleeae (two accessions of *Ligustrum*, one of *Chengiodendron*, *Chionanthus*, *Haenianthus*, *Syringa*, *Priogymnanthus*, *Noronhia*, and *Comoranthus*). For these samples, total genomic DNA was extracted from ca. 5–10 mg of dried leaves. We grounded the samples in 2-mL tubes with three metal beads using a TissueLyser (Qiagen Inc., Texas). We then extracted the DNA following the BioSprint 15 DNA Plant Kit protocol (Qiagen Inc.), and eluted the extracted DNA in 200 µL of AE buffer. Shotgun sequencing (genome skimming approach) was done at the Genopole platform of Toulouse as described in Olofsson et al. [31]. Briefly, 10 to 200 ng of double stranded DNA was used to construct sequencing libraries with the Illumina TruSeq Nano HT Sample kit (Illumina), following the manufacturer’s instructions. DNA was fragmented by sonication, except for extracts from herbarium specimens, which were already highly degraded. Each sample was paired-end sequenced (150 bp) on 1/24th of an Illumina HiSeq3000 lane and multiplexed with samples from the same or different projects.

### 2.2. Assembly of Cytoplasmic and Nuclear DNA Regions

#### 2.2.1. Assembly of Plastome and Nuclear Ribosomal DNA (nrDNA) Cluster

We assembled full plastomes and the nrDNA cluster following the methods of Bianconi et al. [56]. Sequencing depth in these genomic regions was superior to 100× for all investigated species. We generated a consensus sequence for both regions for each accession, and mapped reads onto them with GENEIOUS v9.0.5 [57] for manually checking the assembly quality and assessing the sequencing depth. Then, assembled plastomes and nrDNA clusters were annotated in GENEIOUS by transferring annotations from the olive tree (GenBank accessions NC013707.2 and LR031475.1 for plastid and ribosomal cluster, respectively). Finally, we generated independent alignments for the two regions using the MUSCLE algorithm [58] with default options as implemented in GENEIOUS.

#### 2.2.2. Assembly of Mitochondrial Genes

We adopted a reference-based iterative assembly approach to retrieve a set of 37 mitochondrial protein-coding genes for each sampled species (excluding *Olea europaea*, *Capsicum annuum*, and *Solanum lycopersicum*, for which annotated mitochondrial genomes are already available in GenBank; Table S2). Genes located in regions homologous to plastomes (for which plastid reads mapped on; so called “mtpt” regions) were excluded. Using the reference sequence of the olive tree mitochondrial genes (MG372119.1), an initial set of homologous reads were identified by mapping using Bowtie2 v2.3.5.1 [59] in local mode (all other parameters to default values). These reads were used as the input of a *de novo* assembly using SPAdes v3.14.1 [60] with default parameters. The resulting contigs were then used as reference for the next round of homologous read search and assembly. After three iterations, obtained contigs for each gene were aligned using MAFFT v7.313 [61] with defaults options. Sequencing depth of mitochondrial genes was superior to 30× for all investigated species. The alignment was then inspected and annotated in GENEIOUS by transferring annotations from the olive tree and extremities were trimmed to the annotated coding-sequence.

#### 2.2.3. Assembly of Genes Encoding Phytochromes

Finally, we analyzed phylogenetic relationships within the Oleaceae using a few nuclear phytochrome genes. Their coding part (cds) is relatively long (>3000 bp; 4 exons) and can be aligned on most of their sequence. A reference-guided approach was used to assemble genomic regions containing genes encoding phytochromes B and E (*phyB*, *phyE*), as described in [62,63]. Briefly, raw genomic data sets were filtered using the NGSQC Toolkit v.2.3.3 [64] to retain only high-quality reads (i.e., >80% of the bases with Phred quality score >20), and to remove adaptor contamination and reads with ambiguous bases. The retained reads were subsequently trimmed from the 3’ end to remove bases with Phred score <20. We mapped cleaned paired-end reads on references for genes encoding phytochromes B and E using GENEIOUS. First, exons of *phyB* (two genes, see Phylogenetic analyses below) and *phyE* genes of the ash tree (*Fraxinus excelsior*; GenBank accessions LR983955 to LR983957 [40]) were used as seeds to reconstruct full *phyB* and *phyE* genes of 15 Oleaceae accessions for which nuclear genome sequencing depth was superior or equal to 5× (i.e., *Dimetra craibiana, Nyctanthes arbor-tristis, Abeliophyllum distichum, Forsythia × mandschurica, Fontanesia fortunei, Jasminum didymum, Jasminum pauciflorum, ChrysoJasminum fruticans, Olea europaea* subsp. *laperrinei, Noronhia emarginata, Ligustrum ovalifolium, Syringa pubescens, Schrebera swietenioides, Comoranthus obconicus*, and *Fraxinus ornus*). These species are representative of all main Oleaceae lineages (tribes and subtribes) as defined by Wallander and Albert [3]. We carefully checked that *phy* sequences were not chimeric between related paralogs (especially between *phyB-1a* and *phyB-1b*) by a manual verification of reads phasing on gene assemblies. Then, our newly assembled genes were used to assemble exons in other species by using gene sequences of reference from the same tribe or subtribe. Partial or complete consensus coding sequences of *phyB* and *phyE* were thus obtained for the remaining 46 Oleaceae species. Consensus *phy* sequences of *Ny. arbor-tristis* and *Ch. ligustrinus* showed a relatively high rate of ambiguities on all genes [on average 2.38% (2.26–2.50%) and 2.11% (1.6–2.7%), respectively]. A manual checking of these gene assemblies reveals the presence of more than two distinct homologs suggesting we collapsed sequences of recently duplicated genes on these species. Finally, a few paralogs with lower homology to our references were also detected in some accessions and were further considered when their assembly covered more than 1000 bp of the coding sequence. These additional (pseudo)genes were assembled in nine distantly related species (i.e., *Nor. emarginata, Chionanthus rupicolus, Ch. trichotomus, Fore. angustifolia, Sc. swietenioides, J. didymum, A. distichum, Fors. mandschurica,* and *Fon. fortunei*). Gene sequences covering more than 90% of the coding region were annotated and deposited in GenBank (Table S3). Genes were considered as potentially non-functional when coding sequences were truncated or presented in-frame stop codon.

### 2.3. Phylogenetic Analyses

#### 2.3.1. Phylogeny of Oleaceae Using Organellar DNA

All protein-coding sequences were extracted from the full plastomes and aligned separately as codons using PRANK v170427 [65] (default options for translated alignments of protein-coding DNA sequences). We then estimated a tree using the maximum likelihood (ML) algorithm in IQ-Tree2 v2.0.6 [66]. We used a concatenation approach with an edge-linked proportional partition model, using ModelFinder [67], and assessed branch support with 1000 ultrafast bootstrap (UFB) replicates [68]. The best partition scheme for each dataset was determined with PartitionFinder v2.1.1 [69] and the best fitted evolutionary model for each partition was selected according to the best BIC score with ModelFinder, as implemented in IQ-Tree2. An ML phylogenetic tree for the mitochondrial alignment was also estimated, as described above.

#### 2.3.2. Phylogeny of Oleaceae Using nrDNA

In previous studies on the Oleeae tribe, the nrDNA cluster rendered questionable results with the unexpected phylogenetic clustering of tropical lineages (e.g., Schreberinae subtribe embedded in an Oleinae lineage including genera *Chionanthus, Priogymanthus, Haenianthus, Noronhia*, and *Olea* [30,31,46]). A strong phylogenetic bias was attributed to the highly variable GC content in the external and internal transcribed spacers (ETS and ITS) of the Oleeae tribe [31] and nrDNA was thus deemed unreliable for phylogenetic inference in this group. However, it has been suggested that a purine-pyrimidine only coding (usually referred to as RY-coding) can effectively reduce the influence of biased GC-content [70]. Before using the nrDNA dataset on the phylogenetic analyses, we thus transformed the data from regular nucleotide-coding to a RY-coding alignment. An ML phylogenetic tree was finally estimated as described above splitting the ribosomal cluster into seven partitions: 5’ETS, 18S, ITS1, 5.8S, ITS2, 26S, and 3’ETS.

#### 2.3.3. Phylogenetic Analyses of the Nuclear *phy* Gene Family

Coding regions of all *phy* sequences were aligned together in a matrix using MAFFT (alignment provided in Supplementary Materials). We then estimated a tree for the *phyB*+*phyE* gene family by using the ML algorithm in IQ-Tree2. In this case, we estimated the best substitution model for the whole region using ModelFinder [67], and assessed branch support with 1000 UFB replicates. This analysis allowed us to infer ancestral duplications involved in the diversification of the gene family, and then identify orthologs that can be used for reconstructing phylogeny of Oleaceae. Two nuclear genes (*phyB-1* and *phyE-1*), putatively encoding functional enzymes in most analyzed accessions, were finally selected for the phylogenetic inference of the Oleaceae family. In Oleeae, two paralogs (*phyB-1a* and *phyB-1b*) were kept, with *phyB-1a* arbitrarily aligned to the *phyB-1* copies of other Oleaceae tribes. An ML phylogenetic tree was finally estimated as described above allowing one partition per gene.

#### 2.3.4. Phylogenetic Inference of Family Tree Using Data from Mixed Origin

We then estimated an ML phylogeny for Oleaceae combining nuclear and organellar information and assessed congruence between the datasets by using the algorithm for concordance factors calculations implemented in IQ-Tree2. We quantified the concordance between this phylogeny and each dataset by calculating the gene concordance factor (gCF) and the site concordance factor (sCF) for each branch of the reference tree [71]. The gCF represents the fraction of individual trees (here, species tree obtained with one of the datasets) that is concordant with a given branch, and the sCF shows the proportion of alignment sites that support that branch. It thus allows us to quantify the presence of sites inside each dataset supporting the combined topology, even if the topology obtained with one individual dataset shows an alternative topology.

## 3. Results

### 3.1. Phylogenetic Reconstructions Based on Chloroplast and Mitochondrial Genes

**Figure 1:**
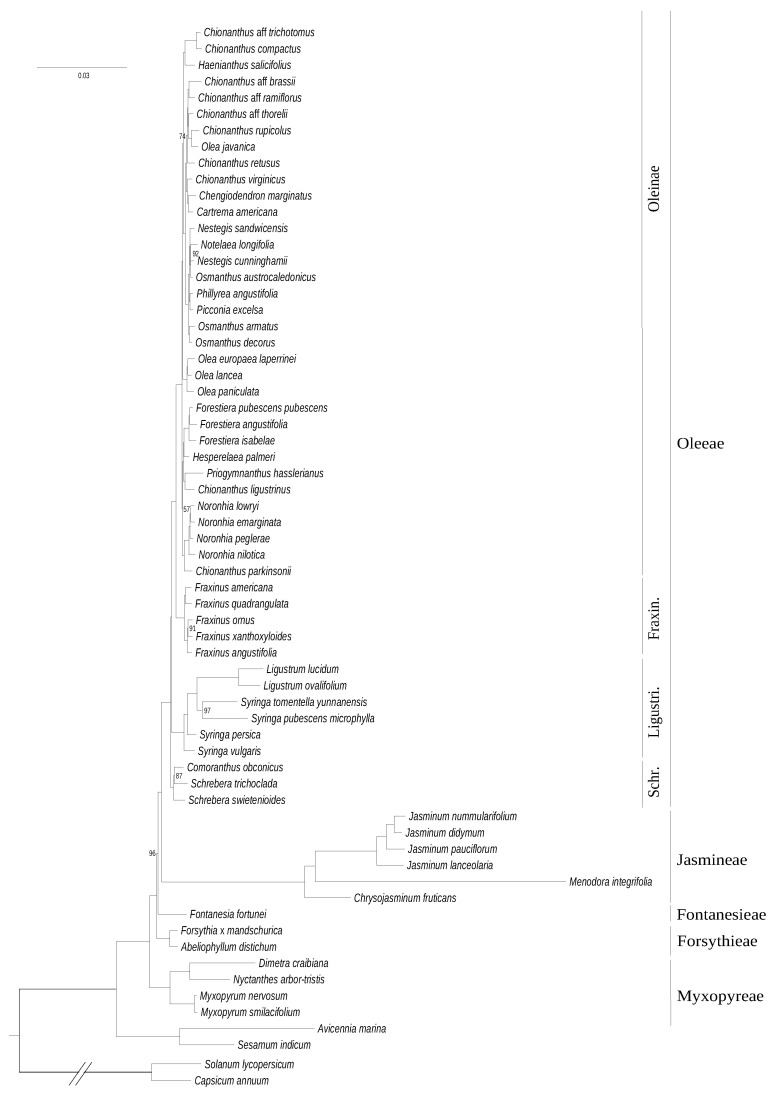
Maximum likelihood phylogenetic tree of Oleaceae based on concatenated coding sequences of 80 plastid genes. The tree was rooted on the split with Solanaceae. The scale is in substitution per site. Ultrafast bootstrap support values are indicated on nodes when inferior to 100.

Using chloroplastic gene data (consisting of 77,676 sites including 10,059 parsimony-informative sites), we obtained a fully-resolved tree of the family (Figure 1). Oleaceae division into five tribes (Myxopyreae, Jasmineae, Forsythieae, Fontanesieae, and Oleeae) is strongly supported. In this dataset, Myxopyreae forms a monophyletic tribe (with the *Myxopyrum* genus sister to *Dimetra*+*Nyctanthes*) and is the sister lineage to all other groups in Oleaceae. Jasmineae appears as sister group to Oleeae. Schreberinae are represented as the sister clade (and subtribe) to the rest of the clades in the monophyletic tribe Oleeae, and *Schrebera* is paraphyletic. Within the Oleeae subtribe Ligustrinae, the genus *Syringa* also forms a paraphyletic group. Within Oleinae, the tree consists of short branches with a few polyphyletic genera (i.e., *Chionanthus, Olea, Osmanthus*, and *Nestegis*). Branch length was particularly long in tribe Jasmineae (notably in *Menodora*) and at a lesser extent in the core Ligustrinae and *Dimetra*+*Nyctanthes*, suggesting an increase of the evolutionary rate of plastid genes in these clades.

**Figure 2:**
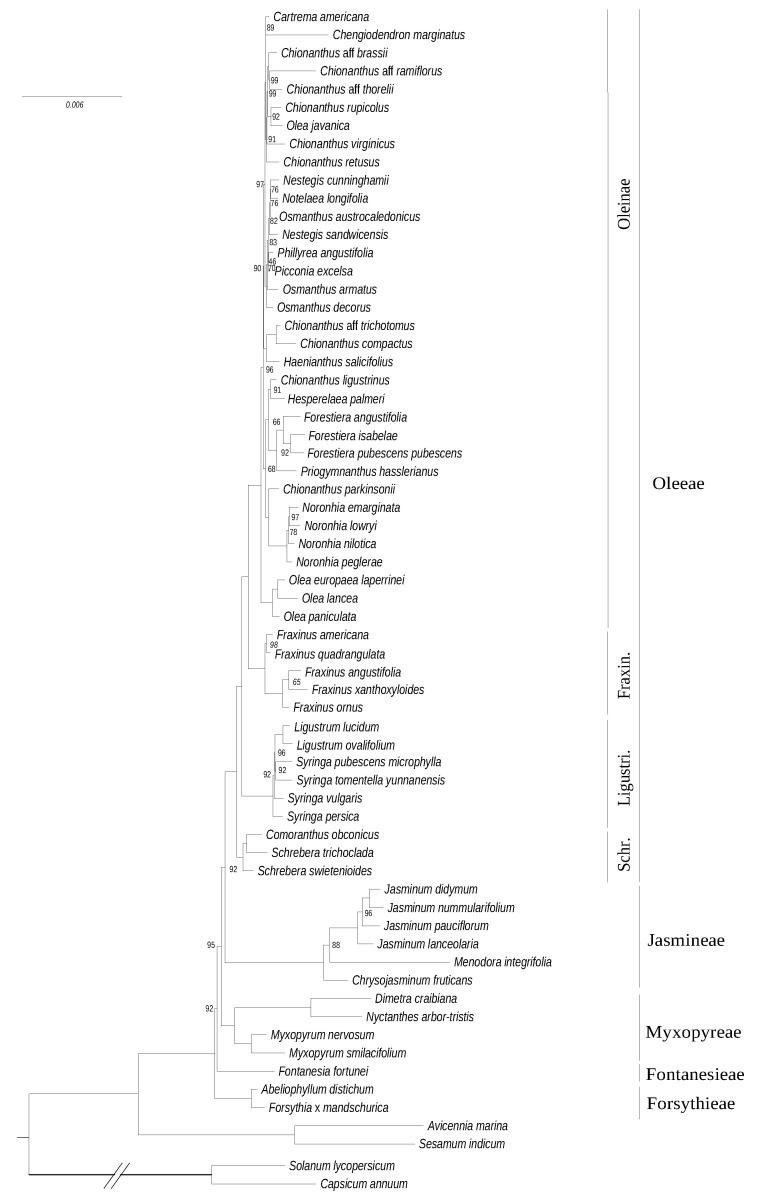
Maximum likelihood phylogenetic tree of Oleaceae based on the concatenation of 37 mitochondrial genes. The tree was rooted on the split with Solanaceae. The scale is in substitution per site. Ultrafast bootstrap support values are indicated on nodes when inferior to 100.

In comparison to the chloroplastic DNA phylogeny, the phylogeny based on mitochondrial data (60,747 sites, 3509 parsimony-informative sites) exhibits a highly-congruent albeit less supported topology (Figure 2). We only stress one significant difference, regarding the branching order in the deepest nodes of the family, in this topology, Forsythieae is positioned as the sister clade to all other Oleaceae (and not Myxopyreae as in the chloroplast tree). Again, Jasmineae (especially *Menodora*) and *Dimetra*+*Nyctanthes* show longer branches suggesting an increase of the evolutionary rate in these two clades.

### 3.2. Phylogeny Based on the Nuclear Ribosomal Cluster

**Figure 3:**
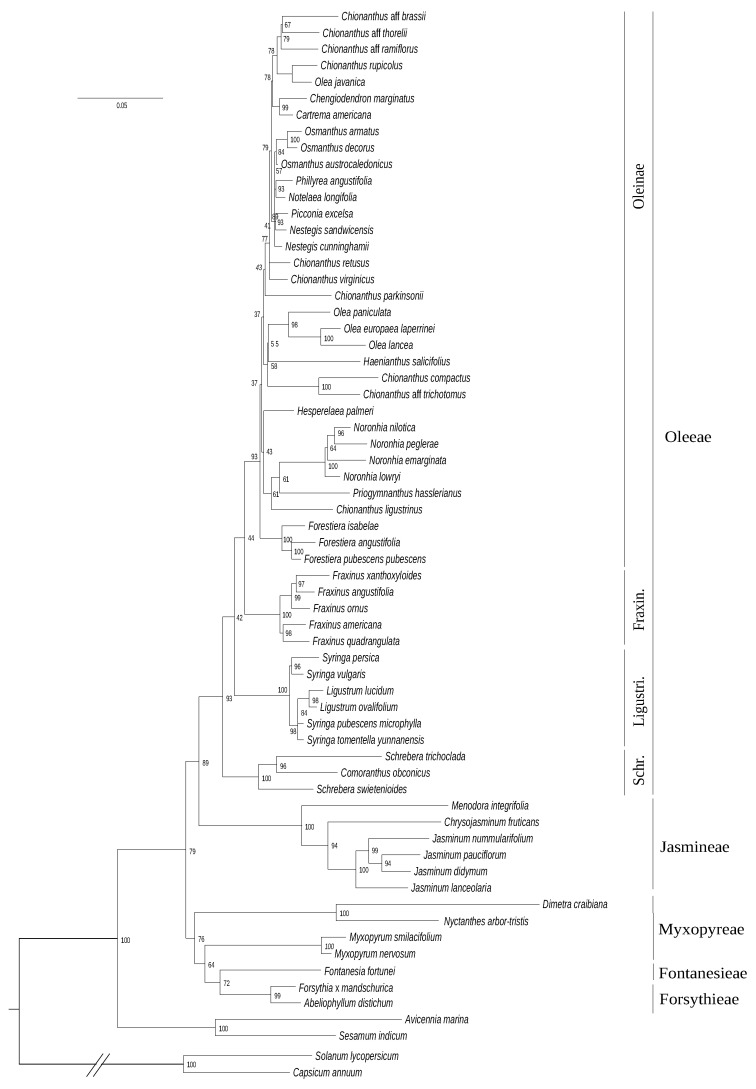
Maximum likelihood phylogenetic tree of Oleaceae based on the complete RY-coded nrDNA cluster alignment. The tree was rooted on the split with Solanaceae. The scale is in substitution per site.

Compared to phylogenetic reconstructions based on cytoplasmic genes, the analysis of the nrDNA cluster (7008 sites, 837 parsimony-informative sites) resulted in a less-supported and quite different topology (Figure 3). Myxopyreae+Fontanesieae+Forsythieae are resolved as sister to the tribes Jasmineae and Oleeae. Myxopyreae are not monophyletic, with *Myxopyrum* sister to *Forsythia+Fontanesia* but this topology is poorly supported (UFB:64). Jasmineae is here again reported as sister to Oleeae but includes a different branching of *Menodora* (sister to *Jasminum*+*Chrysojasminum*). This topology presents a first strongly-supported split in Oleeae between Schreberinae and Fraxininae+Ligustrinae+Oleinae (UFB:97). Within this grouping, Fraxininae and Ligustrinae form monophyletic lineages sister to Oleinae but are not supported. Longer branches are still observed in Jasmineae and *Dimetra*+*Nyctanthes* (especially in *Dimetra*).

### 3.3. Phylogeny Based on Nuclear *phy* Gene Family

**Figure 4:**
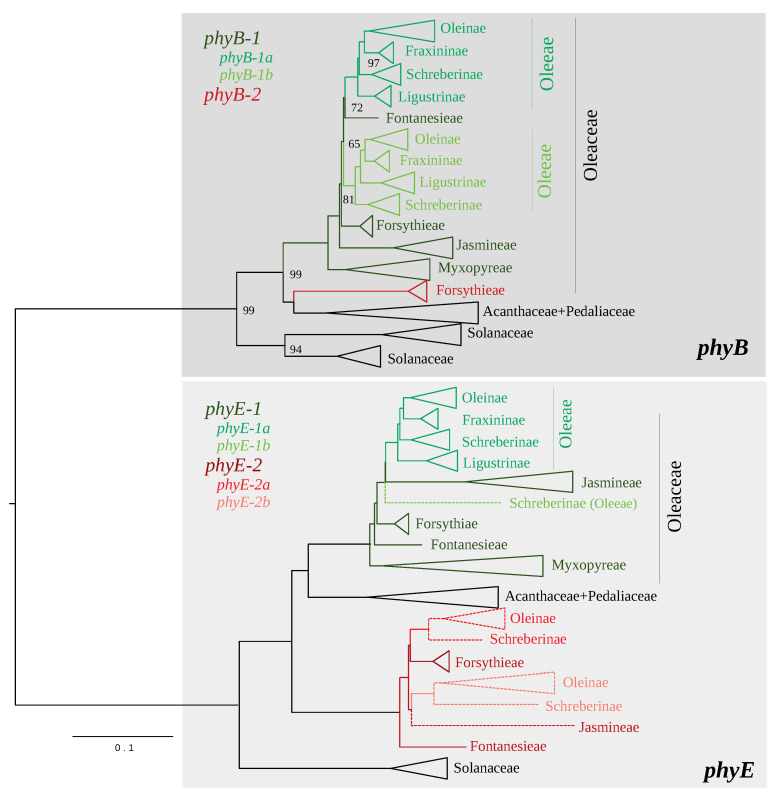
Reduced representation of the midpoint-rooted maximum likelihood phylogenetic tree of the *phy* gene family in Oleaceae. Only ultrafast bootstrap (UFB) values inferior to 100 are indicated on nodes. Putative pseudogenes are denoted by dashed lines.

A second nuclear DNA phylogeny was reconstructed using *phy* genes. We first investigated the phylogenetic tree of the *phy* family in order to select the most informative orthologs. A condensed phylogenetic tree of genes encoding phytochromes E and B is shown in Figure 4 (the detailed tree is provided in Figure S1). As expected, the main distinction of two genes, *phyE* and *phyB*, was recovered.

For *phyE*, one supposedly functional gene (*phyE-1*) was detected in most Oleaceae species, although a second functional gene (*phyE-2*) was also assembled in tribes Forsythieae and Fontanesieae. *phyE-2* is sister to a clade formed by *phyE-1* and *phyE* of *Avicennia* and *Sesamum* (recovered from GenBank). This topology suggests an ancestral gene duplication (giving birth to *phyE-1* and *phyE-2*) in the ancestor of Lamiales, after its divergence from Solanales. A likely pseudogenic *phyE-1* paralog (namely *phyE-1b*) was detected in *Schrebera swietenioides* (Oleeae). Its phylogenetic position remains unresolved due to a polytomy with *phyE-1* clades of Oleeae (namely *phyE-1a*) and Jasmineae. *phyE-1b* likely testifies to a gene duplication in the Oleeae ancestor [3,4], followed by a rapid pseudogenization of this duplicate. Interestingly, we also detected putative pseudogenes of *phyE-2* in distantly related species of Jasmineae and Oleeae. Two putatively pseudogenic lineages were detected in Oleeae (*phyE-2a* and *phyE-2b*), another evidence of (pseudo)gene duplication in the ancestor of this tribe [3,4]. Based on this topology, only *phyE-1* was selected for our phylogenetic analyses of species relationships because this ortholog was detected in all analyzed Oleaceae accessions, and phylogenetic relationships based on this gene support the main taxonomic lineages (i.e., tribes and subtribes) as defined by Wallander and Albert [3]. Putitatively pseudogenized copies (i.e., presence of frame shifts and/or stop codons) of *phyE-1a* were detected in eight species (Figure S1).

For *phyB*, first, two functional duplicates were detected in Solanales, Acanthaceae (*Avicennia*) and Pedaliaceae (*Sesamum*). Two main gene lineages (*phyB-1* and *phyB-2*) were also detected in Oleaceae, but *phyB-2* was detected only in Forsythieae (*Forsythia* and *Abeliophyllum*). This gene is sister to the *phyB* genes of Acanthaceae and Pedaliaceae. On the other hand, *phyB-1* was detected in all Oleaceae species. Two closely related genes (*phyB-1a* and *phyB-1b*) were assembled in all Oleeae species, again testifying to an event of gene duplication in the ancestor of this tribe [3,4]. Based on this topology, *phyB-1* was selected for species relationships analyses because this gene was detected in all analyzed accessions, and the phylogeny allowed us to retrieve all Oleaceae lineages [3]. Putatively pseudogenic copies (i.e., presence of frame shifts and/or stop codons or complete deletion of exon) of *phyB-1a* and *phyB-1b* were detected in two and four species, respectively (Figure S1).

**Figure 5:**
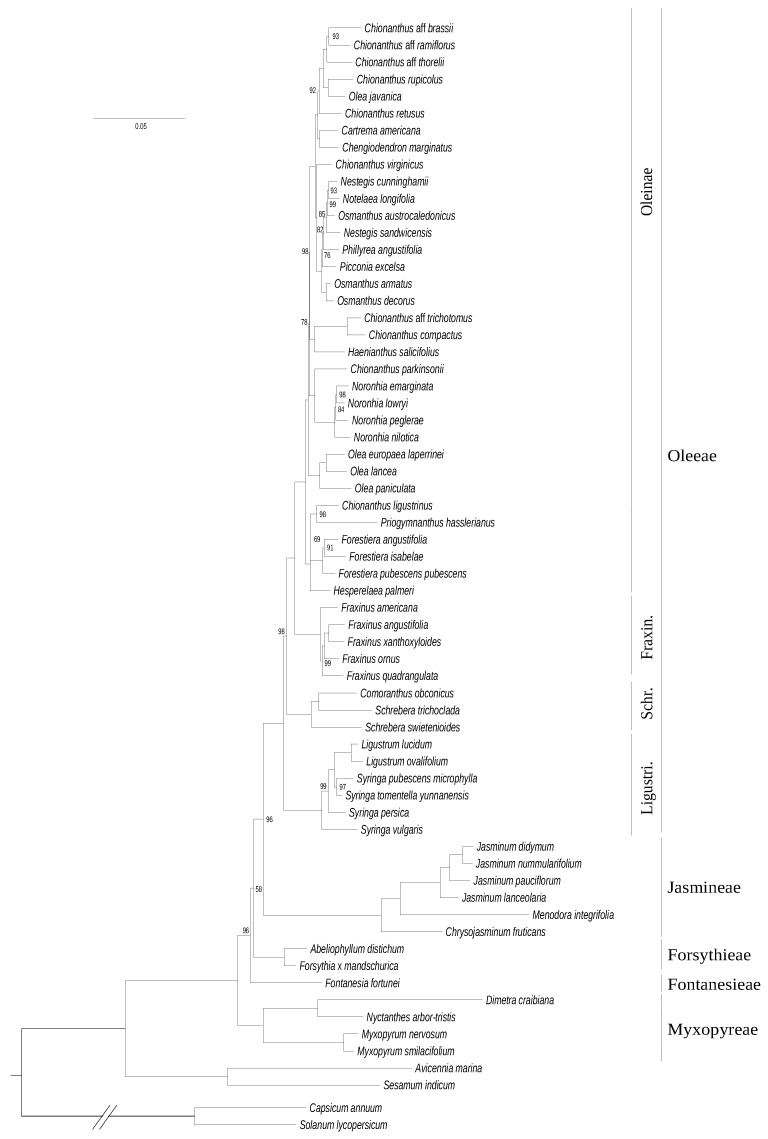
Maximum likelihood phylogenetic tree of Oleaceae based on *phyB-1* (*a* and *b*) and *phyE-1* nuclear genes. Oleeae *phyB-1a* was arbitrarily aligned with *phyB-1* of other tribes. The tree was rooted on the split with Solanaceae. The scale is in substitution per site. Ultrafast bootstrap support values are indicated on nodes when inferior to 100.

The phylogenetic tree based on concatenated *phyB-1* (*a* and *b*) and *phyE-1* genes (10,438 sites, 3282 parsimony-informative sites) is shown in Figure 5. Again, the topology supports the distinction of all taxonomic units defined by Wallander and Albert [3], with tribe Myxopyreae recognized as sister to the rest of Oleaceae. As in other topologies showed above, tribes Jasmineae and Oleeae as well as subtribes Oleinae and Fraxininae are sister groups. In contrast, a major incongruence with both cytoplasmic datasets is the placement of subtribe Ligustrinae as sister to the remaining of Oleeae. This topology was recovered with *phyB-1a* and *phyE-1a*, but not with *phyB-1b* that supports Schreberinae as sister to the other subtribes (Figure 4 and Figure S1). Longer branches are observed in Jasmineae and *Dimetra*.

### 3.4. Phylogenetic Reconstruction Combining the Four Genomic Datasets

**Figure 6:**
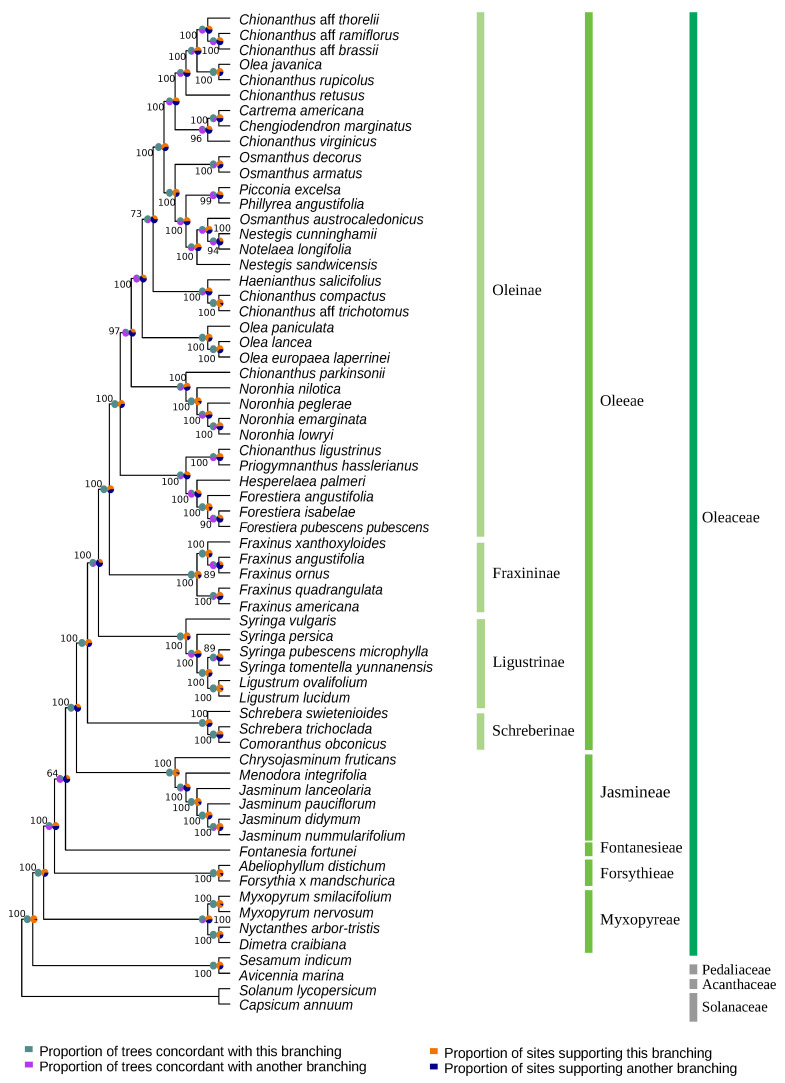
Maximum likelihood topology of Oleaceae family estimated from the partitioned concatenation of 80 plastid coding sequence, 37 mitochondrial genes, the complete nuclear ribosomal DNA cluster and three nuclear genes encoding phytochromes (*phyE-1, phyB-1a, phyB-1b*). Concordance factors were calculated in relation to the species trees inferred for each partitioned dataset. Gene concordance factors are represented by the green/purple pie charts (left), site concordance factors by the blue/orange ones (right). UFB support values are indicated near their respective nodes.

The combination of nuclear and cytoplasmic datasets allowed the reconstruction of a well-supported phylogeny of Oleaceae (Figure 6). All datasets broadly supported the same phylogenetic hypothesis with five strongly supported monophyletic tribes Myxopyreae, Fontanesieae, Forsythieae, Jasmineae and Oleeae. The position of Myxopyreae as sister to the rest of the family is supported by the majority of data as concordance factors attest. The branching order of Forsythieae and Fontanesieae is however difficult to decide on. For these two tribes, the topology of the species tree obtained from the combined dataset is not well-supported. The branching node of Forsythieae, despite a bootstrap support of 100, exhibits high uncertainty based on the concordance factors (gCF: 50%; sCF: 51.4%, Figure S2). The represented branching of Fontanesieae is even less supported (UFB: 64; gCF: 25%; sCF: 29.1%, Figure S2). In both cases, concordance factors show that the reported topology is not supported by most sites. Similar sCF and gCF values suggest this is due to genuine discordant signal in the trees probably due to incomplete lineage sorting. In contrast, we set Jasmineae as the sister tribe of Oleeae with confidence (UFB and gCF values of 100). The topology within Jasmineae confirms the recent reevaluation of the genus *Jasminum* in two distinct genera *Chrysojasminum* and *Jasminum* [36,37,54]. The other major uncertainty resides within the Oleeae tribe on the branching order of Ligustrinae and Schreberinae. Although bootstrap support and concordance factors values sustain the represented branching (Schreberinae as sister to other Oleeae subtribes), the concordance factors (especially sCF) are less decisive for the Ligustrinae split.

## 4. Discussion

We gathered molecular information from several genomic compartments (chloroplastic, mitochondrial and nuclear) for 61 Oleaceae species representative of all currently recognized tribes, subtribes and genera in Oleaceae. Both plastid and mitochondrial DNA datasets as well as the nrDNA cluster are based on relatively high sequencing depth (>30×) and thus of a high quality [30,41]. In contrast, low-copy nuclear genes are more difficult to assemble from genome skimming data and their use in phylogenetics is still a challenge due to lower coverage and recurrent whole genome duplications [31,48]. Here, we explored the utility of a single nuclear gene family (*phyB* and *phyE* genes) for investigating the phylogeny of the whole Oleaceae family. The obtained dataset allowed us to tackle the complex history of nuclear gene duplication and subsequent pseudogenization indicating the necessity to control for gene orthology before proposing a phylogenetic hypothesis for the whole family. By combining and confronting our datasets, we were able to establish a well-resolved phylogeny of Oleaceae although a few discordances were revealed when comparing phylogenies based on cytoplasmic and nuclear genomic regions. Overall, tribes and subtribes were strongly supported by all phylogenetic reconstructions and only very few relationships between tribes/subtribes were not fully resolved.

### 4.1. Taxonomy of Oleaceae

Our phylogenetic analyses confirm the divisions of Oleaceae in five tribes and four subtribes as defined by Wallander and Albert [3]. Given the amount of data we analyzed, we achieved a greater resolution and support in our phylogenetic inference of the whole family, including all currently recognized genera and considering several accessions from distant areas in the largest groups (e.g., *Chionanthus, Olea, Fraxinus, Syringa, Jasminum*). First, our results validated the grouping of *Nyctanthes, Dimetra* and *Myxopyrum* in Myxopyreae [3,72] and overall supported this clade as sister to all other lineages in the family. We were also able to corroborate some of the less-reliable nodes and in particular the sister tribes Jasmineae and Oleeae. We resolved the relationships between Forsythieae and Fontanesieae as being distinct and non-sister tribes. We also put into question the idea that Ligustrinae is sister to all other lineages in Oleeae [3,35,36] favoring the alternative hypothesis of Schreberinae being the one (as in [31], where the whole plastid genome and single-nucleotide polymorphisms datasets gathered from more than 11,000 nuclear genes were used). Finally, we were also able to better define the relationships within Oleeae wherein some genera appeared as polyphyletic (i.e., *Chionanthus, Olea, Osmanthus, Nestegis*) or paraphyletic (i.e., *Schrebera*, *Syringa*) confirming previous reports from the literature [26,28,30,31,32].

A relatively high congruence was obtained between phylogenies based on plastid and mitochondrial DNA datasets (Figure 1 and Figure 2), as expected for maternally inherited genomes [42]. We obtained the best resolution with the chloroplastic dataset as it contains more informative sites. Topologies based on *phy* genes and cytoplasmic genomes were also quite congruent although the relative placement of Ligustrinae and Schreberinae as well as Forsythieae and Fontanesieae differ according to *phy* genes (Figure 4 and Figure 5). In contrast, the nrDNA cluster provided less reliable information than organellar genomes and *phy* nuclear genes (Figure 3). Phylogenetic biases related to GC content and incomplete concerted evolution have been already reported in Oleaceae for the nrDNA marker (e.g., [10,31,46]), which thus needs to be interpreted with caution. Yet, the RY-coding seems to have greatly improved the topology since all Oleeae subtribes were retrieved in contrast to previous analyses [31,46] (see Figure S3 for the ML phylogeny from the original alignment).

### 4.2. Nuclear Gene Orthology and Polyploidization Events in Oleaceae

The analysis of a small multigene family revealed other aspects on the Oleaceae history, related to past whole genome duplications and different tempo of pseudogenization. First, two divergent functional paralogs were revealed on *phyE* and *phyB*, but only in Fontanesieae and Forsythieae. The duplication of these genes (possibly due to whole genome duplication) is ancient, likely preceding the divergence of Lamiales, and the pseudogenisation of *phy-B2* and *phy-E2* in tribes Myxopyreae, Jasmineae and Oleeae may have occurred rapidly after their divergence. Only pseudo-*phy-E2* was still detected in Jasmineae and Oleeae. More interestingly, the detection of two closely related paralogs of *phyB-1*, *phyE-1* and pseudo-*phyE-2* in all Oleeae species is highly congruent with the reported event of polyploidization in their common ancestor [3,4]. As we decided to collapse highly homologous sequences of *phy* genes, we were not able to investigate the fate of these genes in neopolyploids, but we detected a relatively high level of ambiguities in the tetraploid *Ny. arbor-tristis* [73] as well as in *Ch. ligustrinus* for which the chromosome number is unknown.

## 5. Concluding Remarks and Future Directions in Oleaceae Phylogenomics

Our work provided a more robust phylogenetic history of Oleaceae than previous works, a crucial prerequisite to study the diversification process of this family. A complex history of gene duplication and pseudogenization was also revealed, and these aspects need to be evaluated before using nuclear data in the reconstruction of phylogenies, especially in a plant family with paleopolyploids such as Oleaceae. Moreover, our prospective study also demonstrated the limits of using *phy* genes to estimate a tree due to the variable levels of gene retention and the presence of non-functional sequences. With the higher accessibility of genomic data, some of these caveats can be circumvented with the use of new methodologies such as the analyses of UCE (Ultra Conserved Elements) or universal single-copy orthologs (e.g., [74,75,76]). Although, in the light of the complicated history of evolution of plants (e.g., multiple reported events of whole genome duplication), we stress the importance of taking gene orthology into account when estimating species trees.

When it comes to our current and future goals with the study of the phylogenomics of Oleaceae, the complete sequencing of nuclear genomes (with at least 30–50× coverage) is in progress in our lab. We are mainly focusing on low heterozygous diploid species, and avoiding neo-polyploids and hybrids. In addition, since this study confirmed that cytoplasmic and nuclear ribosomal DNA sequences can be easily assembled independent of species ploidy, we are using those genomic regions on a comprehensive sampling to reconstruct a fossil-calibrated phylogeny of the family. Finally, with this large phylogeny of Oleaceae we will explore the causes of variable evolutionary rates among genomes, considering factors as generation time (e.g., short living species exhibit particularly long branches in phylogenetic reconstructions) [77], gene duplication, genome inheritance, and recombination rate [77,78,79].

## Supplementary Materials

The following are available online at https://www.mdpi.com/2073-4425/11/12/1508/s1. Table S1. List of Oleaceae accessions analyzed in our study, with their taxonomy, accession number and origin. Table S2. List of species used as outgroups in our phylogenetic analyses. Table S3. GenBank no of genomic regions for each accession. Figure S1. Full representation of the midpoint-rooted maximum likelihood phylogenetic tree of the *phy* gene family in Oleaceae. Figure S2. Maximum likelihood topology of Oleaceae estimated from the partitioned analysis of the four datasets with corresponding concordance factors of nodes. Figure S3. Maximum likelihood phylogenetic tree of Oleaceae based on the non-transformed nrDNA cluster alignment. Materials S1 to S11. Sequence alignments used for phylogenetic reconstructions, and tree files.
