# Supplementary material for: Resolving the Phylogeny of the Olive Family (Oleaceae): Confronting Information from Organellar and Nuclear Genomes
Source: Genes (Basel). 2020 Dec 16;11(12):1508. doi: 10.3390/genes11121508 (PMC7767060; doi:10.3390/genes11121508)
Supplement: Supplementary file 1 [file genes-11-01508-s001.zip › Supplementary_Information_R1.docx]

**Supplementary Information**

**Resolving the phylogeny of the olive family (Oleaceae): confronting information from organellar and nuclear genomes**

Julia Dupin & Pauline Raimondeau, *et al.*

**Supplementary information includes the following items:**

**Table S1**. List of Oleaceae accessions analyzed in our study, with their taxonomy, accession number and origin

**Table S2**. List of species used as outgroups in our phylogenetic analyses

**Table S3.** GenBank no of genomic regions for each accession

**Figure S1.** Full representation of the midpoint-rooted maximum likelihood phylogenetic tree of the *phy* gene family in Oleaceae

**Figure S2.** Maximum likelihood topology of Oleaceae estimated from the partitioned analysis of the four datasets with corresponding concordance factors of nodes.

**Figure S3.** Maximum likelihood phylogenetic tree of Oleaceae based on the non-transformed nrDNA cluster alignment

**Supplementary materials.** Sequence alignments used for phylogenetic reconstructions: 1. Complete alignment of *phy* genes in phylip format; 2. Concatenated alignment of *phyB-1* and *phyE-2* genes annotated in nexus format; 3. Complete annotated alignment of plastid coding sequences in nexus format; 4. Complete annotated alignment of mitochondrial genes in nexus format; 5. Complete annotated alignment of nrDNA sequences in nexus format. 6. ML plastid tree with bootstrap values in newick format. 7. ML mitochondrial tree with bootstrap values in newick format 8. ML RY-coded nrDNA with bootstrap values in newick format. 9. ML phy tree with bootstrap values in newick format. 10. ML tree of *phyB-1* and *phyE-2* genes with bootstrap values in newick format. 11. ML tree of concatenated dataset with bootstrap, gCF and sCF values in newick format.

**Table S1**. List of Oleaceae accessions analyzed in our study, with their taxonomy, accession number and origin (including country and collection). *N = naturalized plant in the introduced range; BG = Botanical Garden; CEFE = Centre d'Ecologie Fonctionelle et Evolutive, Montpellier; Herbarium acronyms follow Thiers (2019); ^h^ Sample directly removed from the herbarium specimen. ^n^ Newly analyzed accession.

| **Tribe (Subtribe)** | **Species** | **Accession no/cultivar name** | **Country (Collection)** |
| --- | --- | --- | --- |
| Myxopyreae | *Dimetra craibiana* Kerr | S. Suddee et al. A. Paton, T. Jonganurak & V. Chamchumroon 2014 (K)^n^ | Thailand |
|  | *Myxopyrum* *nervosum* Blume | S.C. Chin 13831 [P04255167]^h, n^ | Pahang, Malaysia |
|  | *Myxopyrum smilacifolium* (Wall.) Blume | J.E. Vidal 6056 [P03424472]^h, n^ | Nam Ngum, Vietnam |
|  | *Nyctanthes arbor-tristis* L. | C. Parma 9713 (M)^h, n^ | Xxx, India |
| Fontanesieae | *Fontanesia fortunei* Carrière | T. Joẞberger 1829 (BONN) | China (Bonn BG) |
| Forsythieae | *Abeliophyllum* *distichum* Nakai | M.W. Chase 3881 (K) | Korea (Kew BG) |
|  | *Forsythia* *×* *mandschurica* Uyeki | 18-40-1968 (JFRU21) | China (Montreal BG) |
| Jasmineae | *Chrysojasminum fruticans* (L.) Banfi | G. Besnard 01-2016 (P)^n^ | CEFE Montpellier, France (CEFE) |
|  | *Jasminum didymum* G.Forst. | L. Barrabe 1312 [P01062395]^h, n^ | Boulouparis, New Caledonia |
|  | *Jasminum lanceolaria* Roxb. | L. Jin-Kui 794 [P04254370]^h, n^ | Mt. Shunhuangshan, China |
|  | *Jasminum nummularifolium* Baker | J.N. Labat 3712 [P00527351]^n^ | Ngoumi, Comoro |
|  | *Jasminum pauciflorum* Benth. | D. Bilivogui 193 [P00853672]^h, n^ | Mts Nimba, Guinea |
|  | *Menodora integrifolia* (Cham. & Schltdl.) Steud. | A.L. Cabrera 29169 [P04492682]^h, n^ | dep. Apostoles, Argentina |
| Oleeae (Fraxininae) | *Fraxinus americana* L. | 968-79 (R.E. Weaver & J. Nickerson) | USA (Arnold Arboretum) |
|  | *Fraxinus angustifolia* Vahl | G. Besnard 42-2014 (P) | INRA Montpellier, France |
|  | *Fraxinus ornus* L. | Cefe-B2 (P. Saumitou-Laprade) | CEFE, France (CEFE) |
|  | *Fraxinus quadrangulata* Michx. | 14654 (G.W. Letterman) | USA (Arnold Arboretum) |
|  | *Fraxinus xanthoxyloides* (G.Don) Wall. ex A.DC. | Kepos 45350 (K) | Afghanistan (Kew BG) |
| Oleeae (Ligustrinae) | *Ligustrum lucidum* W.T.Aiton | G. Besnard 01-2019 (P)^n^ | La Croix Falgarde, France (N*) |
|  | *Ligustrum ovalifolium* Hassk. | G. Besnard 01-2018 (P)^n^ | Portugal (CEFE Montpellier, CCEFE, N*) |
|  | *Syringa persica* L. | cv. "Laciniata"^n^ | Unknown (cultivated) |
|  | *Syringa* *pubescens* Turcz. subsp. *microphylla* (Diels) M.C.Chang & X.L.Chen | cv. "Superba" / G. Besnard 02-2015 (P) | China (Toulouse BG) |
|  | *Syringa tomentella* Bureau & Franch. subsp. *yunnanensis* (Franch.) J.Y.Chen & D.Y.Hong | R. Lancaster 934 | China (Arnold Arboretum) |
|  | *Syringa vulgaris* L. | G. Besnard 02-1997 (P) | Sedenay, France |
| Oleeae | *Comoranthus obconicus* Knobl. | C. Mas 98 [P00176499]^h, n^ | Ilot Choazil, Mayotte |
| (Schreberinae) | *Schrebera* *swietenioides* Roxb. | W.S. Kurz 2312 (K)^h^ | Myanmar |
|  | *Schrebera trichoclada* Welw. | P.J. Greenway et al. 15382 (MO)^h^ | Tanzania |
|  |  |  |  |

**Table S1, end**.

| **Tribe (Subtribe)** | **Species** | **Accession no/cultivar name** | **Country (Collection)** |
| --- | --- | --- | --- |
| Oleeae (Oleinae) | *Cartrema americana* (L.) Raf. | CHW-OA (MO) | Xxx, USA |
|  | *Chengiodendron marginatus* (Champ. ex Benth.) C.B.Shang *et al.*, X.R.Wang, Y.F.Duan & Y.F.Li | J.P.W. Woo 138 [P04074276]^h, n^ | Pat Sin Range, Hong Kong, China |
|  | *Chionanthus* aff. *brassii* (Kobuski) Kiew | J.F. Molino 3078 (MPU) | Xxx, Papua New Guinea |
|  | *Chionanthus compactus* Sw. | C.M. Taylor 8582 [P03868414]^h, n^ | Mpio. de Toa Baja, Puerto Rico |
|  | *Chionanthus ligustrinus* (Sw.) Pers. | A.H. Liogier 15164 [P03384280]^h^ | Jaiqui Picado, Dominican Republic |
|  | *Chionanthus parkinsonii* (Hutch.) Bennet & Raizada | M. Van de Bult 1222 (M)^h^ | Xxx, Thailand |
|  | *Chionanthus* aff. *ramiflorus* Roxb. | M.E. Polane 24397 (K)^h^ | Xxx, Vietnam |
|  | *Chionanthus retusus* Lindl. & Paxton | coll. KEW-13008 | China (Kew BG) |
|  | *Chionanthus rupicolus* (Lingelsh.) Kiew | W. Takeuchi et al. 15149 (K)^h^ | Xxx, Papua New Guinea |
|  | *Chionanthus* aff. *thorelii* (Gagnep.) P.S.Green | M.F. Newman 2145 [P00577947] | Prey Kravanh, Cambodia |
|  | *Chionanthus* aff. *trichotomus* (Vell.) P.S.Green | St.G. Beck 25118 (M)^h^ | BXxx, Bolivia |
|  | *Chionanthus virginicus* L. | coll. KEW-1976292 | USA (Kew BG) |
|  | *Forestiera angustifolia* Torr. | R. Kathy 1861, coll. 1997-0035-100 | Xxx, USA |
|  | *Forestiera isabelae* Hammel & Cornejo | de Hammel & Perez 24248 (K)^h^ | Xxx, Costa Rica |
|  | *Forestiera pubescens* Nutt. var. *pubescens* | LBJWC-0204 | USA |
|  | *Haenianthus salicifolius* Griseb. | A.H. Liogier 12182 [P04255168]^h, n^ | La Rucilla, Dominican Republic |
|  | *Hesperelaea palmeri* A.Gray | E. Palmer 81 (MO)^h^ | Guadalupe, Mexico |
|  | *Nestegis* *cunninghamii* (Hook.f.) L.A.S.Johnson | Kepos 45352 (K), coll. KEW-1966-67114 | Xxx, New Zealand |
|  | *Nestegis sandwicensis* (A.Gray) O.Deg., I.Deg. & L.A.S.Johnson | T. Flynn 6329 (MPU) | Hawaii, USA |
|  | *Noronhia emarginata* (Lam.) Poir. | T. Flynn 6331 (MPU) | HSt Philippe, Hawaii, USA (N*) |
|  | *Noronhia lowryi* Hong-Wa | J. Razanatsoa 686-1 (TAN) | Itremo, Madagascar |
|  | *Noronhia nilotica* (Oliv.) Hong-Wa & Besnard | White 886 (MO)^n^ | Xxx, Gabon |
|  | *Noronhia peglerae* (C.H.Wright) Hong-Wa & Besnard | O. Maurin 1766 (PET) | Xxx, South Africa |
|  | *Notelaea longifolia* Vent. | L.A. Craven et al. 10154 [P04255187]^h^ | Pebbly Beach, Australia |
|  | *Olea europaea* L. subsp. *laperrinei* (Batt. & Trab.) Cif. | coll. CEFE-Adjelella 10 | Adjellela, Hoggar, Algeria (CEFE) |
|  | *Olea javanica* (Blume) Knobl. | G. Besnard s.n. (MPU) | Xxx, Indonesia |
|  | *Olea lancea* Lam. | G. Besnard s.n. (P) | Xxx, Reunion |
|  | *Olea paniculata* R.Br. | C. Lambrides 1 (MPU) | Brisbane, Australia |
|  | *Osmanthus armatus* Diels. | G. Besnard 02-2013 (P) | China (Toulouse BG) |
|  | *Osmanthus* *austrocaledonicus* (Vieill.) Knobl. | J.K. Munzinger 1662 [P00354333] | Mt Kouakoué, New Caledonia |
|  | *Osmanthus decorus* (Boiss. & Balansa) Kasapligil | Accession Z-1 | Caucasus (KEITH arboretum) |
|  | *Phillyrea angustifolia* L. | Restinclières | Prades de Lez, France |
|  | *Picconia excelsa* (Aiton) DC. | PT-0-BR-20110182-45 | Madeira, Portugal |
|  | *Priogymnanthus hasslerianus* (Chodat) P.S.Green | T. Rojas 10694 [P03384284]^h, n^ | Sierra de Amambay, Paraguay |
|  |  |  |  |

**Table S2**. List of species used as outgroups in our phylogenetic analyses. Number of genome project, accession nos and DNA regions are indicated.

| **Family** | **Species** | **Genome no (Plant isolate)** | **Accession no (DNA region)** |
| --- | --- | --- | --- |
| Acanthaceae | *Avicennia marina* (Forssk.) Vierh. | PRJNA629068 (RG18007) | CM0231175 (*phyB*), CM0231176 (*phyB*), CM023179 (*ΨphyE*) |
|  |  | PRJNA628464 | NC_047414.1 (plastome) |
|  |  | PRJNA6290689 | SRR11912464 (mtDNA/nrDNA) |
| Pedaliaceae | *Sesamum indicum* L. | PRJNA268358 (Zhongzhi no 13) | XM_011073075 (*phyB*), XM_011102453 (*phyB*), XM_011086986 (*phyE*), SRR1055197 (mtDNA/nrDNA) |
|  |  | PRJNA78529 | NC_016433.2 (plastome) |
| Solanaceae | *Capsicum annuum* L. | PRJNA186921 | CA05g16200 (*phyB*), CA01g16010 (*phyB*), CA02g12340 (*phyE*), JX270811.1 9 (plastome), KJ865410.1 (mtDNA), SRR653499 (nrDNA) |
| Solanaceae | *Solanum lycopersicum* L. | PRJNA119 | Solyc05g053410 (*phyB*), Solyc01g059870 (*phyB*), Solyc02g071260 (*phyE*), DQ347959.1 (plastome), MF034192.1 (mtDNA), NW_020442480.1 (nrDNA) |


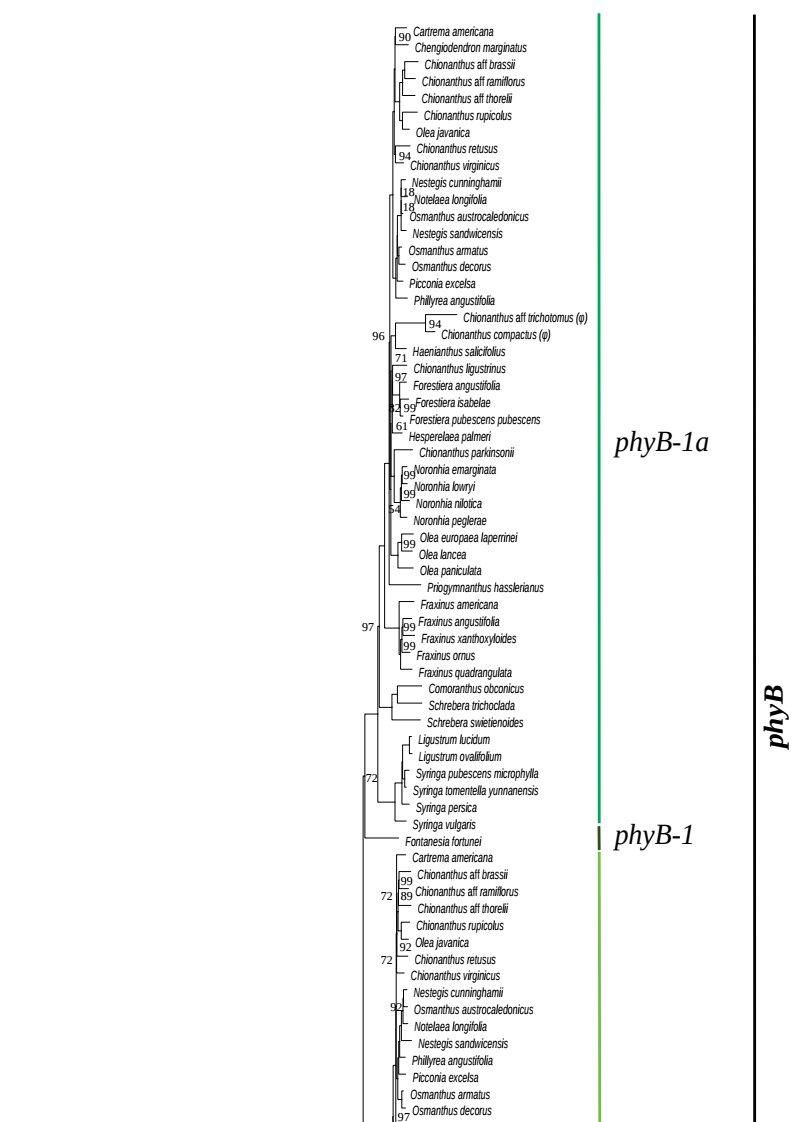
**Figure S1.** Full representation of the midpoint-rooted maximum likelihood phylogenetic tree of the *phy* gene family in Oleaceae. Only ultrafast bootstrap values inferior to 100 are indicated on nodes.


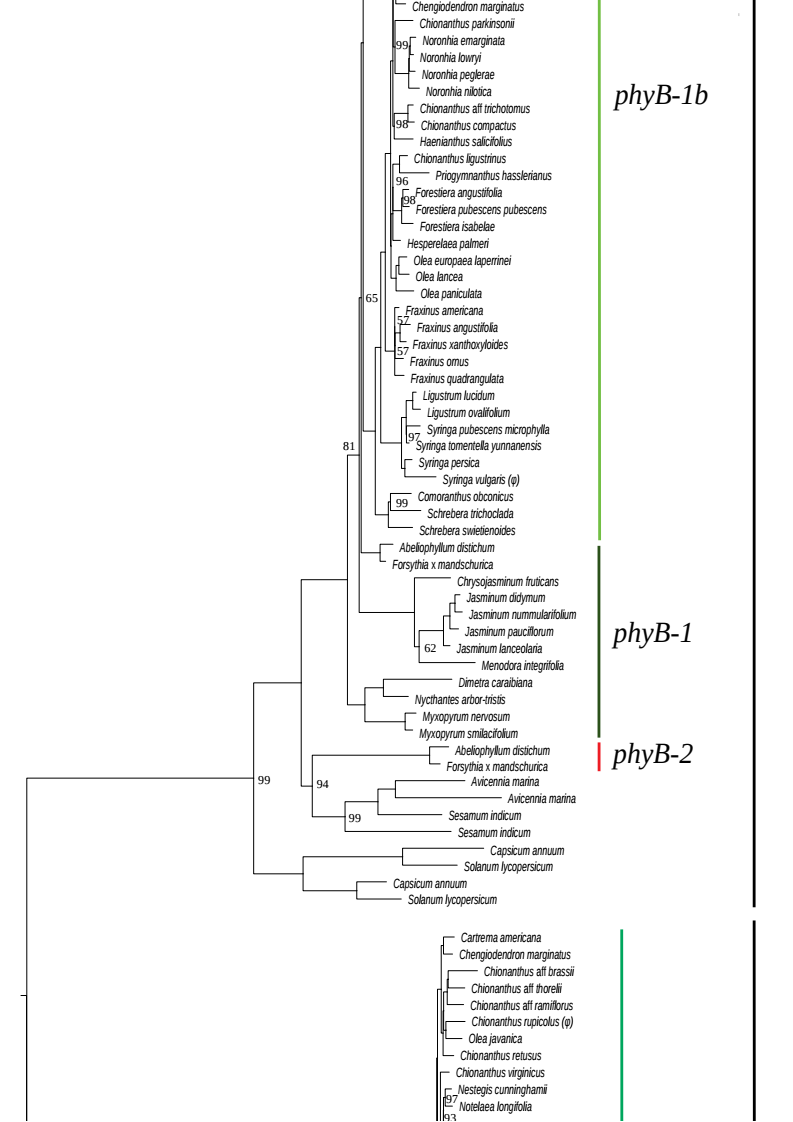
**Figure S1,**  continued.


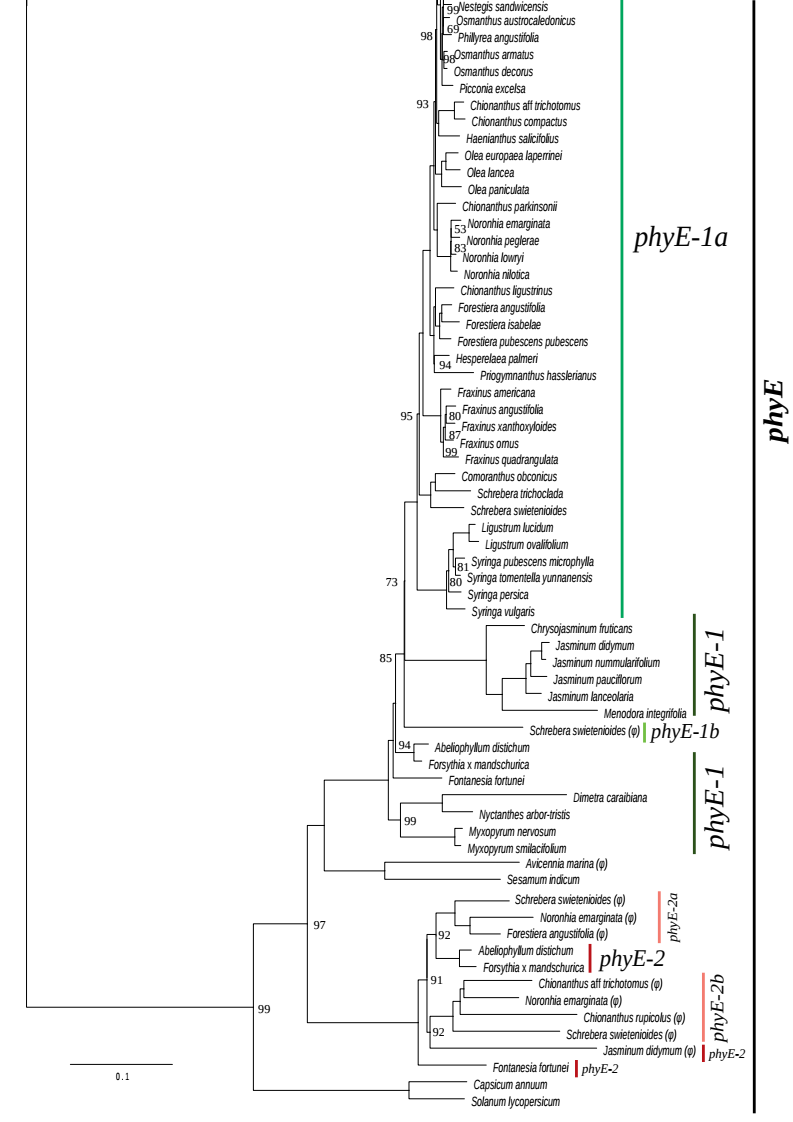
**Figure S1,** end.

**
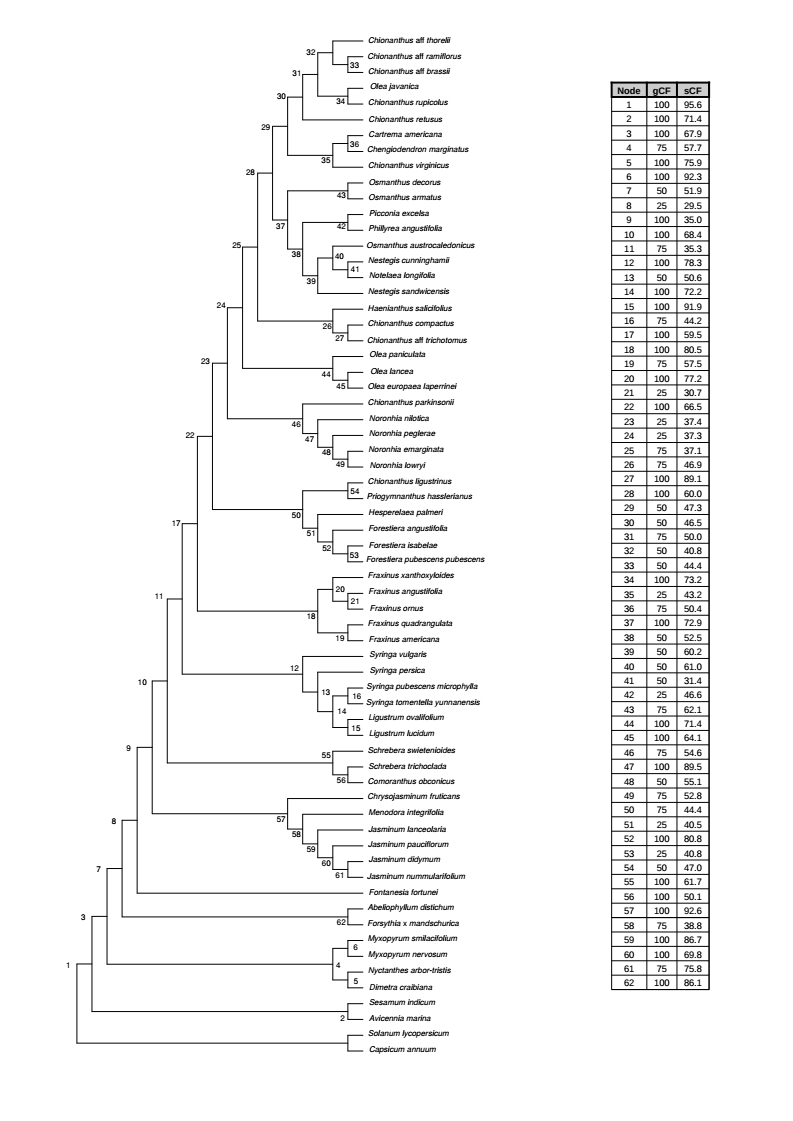
Figure S2.** Maximum likelihood topology of Oleaceae estimated from the partitioned analysis of the four datasets with corresponding concordance factors of nodes. Each node is labeled with a number used as a reference to their respective concordance values in the table.


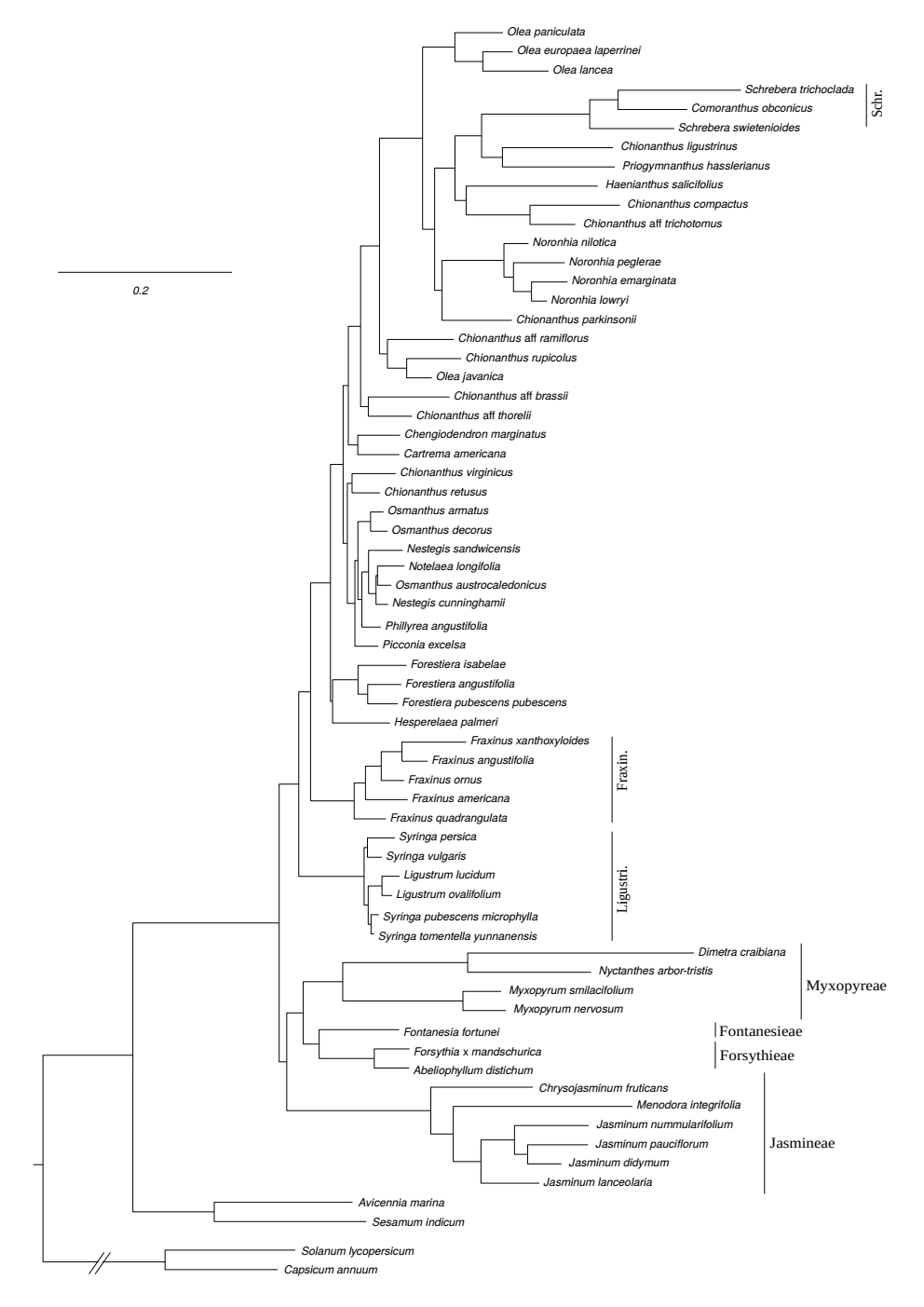
**Figure S3.** Maximum likelihood phylogenetic tree of Oleaceae based on the non-transformed nrDNA cluster alignment. The tree was rooted on the split with Solanaceae.

**Reference**

Thiers B (2019) Index Herbariorum: A global directory of public herbaria and associated staff. New York Botanical Garden’s Virtual Herbarium. *http://sweetgum.nybg.org/science/ih/* [accessed: 16 September 2020]
